# Bedford County Hospital

**Published:** 1912-03-30

**Authors:** 


### BEDFORD COUNTY HOSPITAL.

Mr. Edwin Ransom, of Bedford, J.P., an ex-P.L.G., and formerly on the Bedford County Hospital Board, writes: You recently commented on two departments of our County Hospital—the laundry and the *x*-ray. The scientific house surgeon is, as you say, naturally supported by the medical staff in the desire that the latter should be thoroughly refitted, a small thing compared with a new laundry, which is estimated at £600 or more. The kernel of any laundry is the washing-machine, and in a hospital laundry its importance is obviously supreme. Lord Lister introduced purity into surgery. Let us carry out the principle in hospital laundries. Under the term "foul-linen washer" laundry engineers supply the ordinary rotary machine, provided with specially fitted doors, inlets and outlets, which preclude the handling of articles during the process, also the possibility of smell. Three waters for swilling, cleansing (with the usual detergents), and rinsing succeed each other. On opening the machine the contents are transferred to the hydro-extractor or other arrangement for drying. Such a machine was fixed a year ago at our large Workhouse at a cost of less than £60. Decency, health, and £2 in wages, etc., have been saved weekly, and imbeciles are no longer depended on to do any of the work. With such testimony from several other Workhouses one may regret that such a machine is not in universal use at hospitals, whether the rest of the laundry is up to date or not.

